# Lectin-mediated protocell crosslinking to mimic cell-cell junctions and adhesion

**DOI:** 10.1038/s41598-018-20230-6

**Published:** 2018-01-31

**Authors:** Sarah Villringer, Josef Madl, Taras Sych, Christina Manner, Anne Imberty, Winfried Römer

**Affiliations:** 1 Faculty of Biology, Albert-Ludwigs-University Freiburg, Schänzlestraße 1, 79104 Freiburg, Germany; 2 Bioss - Centre for Biological Signalling Studies, Albert-Ludwigs-University Freiburg, Schänzlestraße 18, 79104 Freiburg, Germany; 3 Freiburg Center for Interactive Materials and Bioinspired Technology (FIT), Albert-Ludwigs-University Freiburg, Georges-Köhler-Allee 105, 79110 Freiburg, Germany; 4 Laboratoire de Bioimagerie et Pathologies, UMR 7021 CNRS, Faculté de Pharmacie, Université de Strasbourg, 67401 Illkirch Cedex, France; 5 CNRS, CERMAV, Univ. Grenoble Alpes, 38000 Grenoble, France; 6 Present Address: Focal Area of Infection Biology, Biozentrum, University of Basel, 4056 Basel, Switzerland

## Abstract

Cell adhesion is a crucial feature of all multicellular organisms, as it allows cells to organise themselves into tissues to carry out specific functions. Here, we present a mimetic approach that uses multivalent lectins with opposing binding sites to crosslink glycan-functionalised giant unilamellar vesicles. The crosslinking process drives the progression from contact puncta into elongated protocellular junctions, which form the vesicles into polygonal clusters resembling tissues. Due to their carbohydrate specificity, different lectins can be engaged in parallel with both natural and synthetic glycoconjugates to generate complex interfaces with distinct lectin domains. In addition, the formation of protocellular junctions can be combined with adhesion to a functionalised support by other ligand-receptor interactions to render increased stability against fluid flow. Furthermore, we consider that adhesion is a complex process of attraction and repulsion by doping the vesicles with a PEG-modified lipid, and demonstrate a dose-dependent decrease of lectin binding and formation of protocellular junctions. We suggest that the engineering of prototissues through lectin-glycan interactions is an important step towards synthetic minimal tissues and in designing artificial systems to reconstruct the fundamental functions of biology.

## Introduction

Synthetic biology combines the expertise from diverse disciplines and accordingly comes with different visions^1,2^. One aim is to engineer minimal cells from a bottom-up approach in order to redesign life. As membrane-enclosed aqueous compartments are key features of all biological cells, giant unilamellar vesicles (GUVs) are considered as protocells which can be constituted with features to support e.g. cell homeostasis, self-reproduction, and other features that are perceived necessary to be “living”^3,4^. A successive aim of the generation of protocells should be the construction of prototissues, as in all multicellular organisms cells contact and interact with each other in order to carry out specific functions.

In the biotechnological field, multicompartment liposome clusters, generated through asymmetric nucleic acid binding, present promising research for the generation of multi-step chemical processes within liposomal modules^5–7^. Besides, the use of giant vesicles allows to identify different adhesive and anti-adhesive agents e.g. biotin-streptavidin^8,9^, polymers^10^, or peptides^11^. In order to understand the origin of life, giant vesicle “colonies” generated by vesicle aggregation through electrostatic attraction are used to answer the question whether cell communities have had advantages compared to isolated cells^12,13^. In nature, however, many different cell adhesion proteins are involved to generate interactions between cells, or between cells and the extracellular matrix. Cell surface-proteins like the cell adhesion molecules (CAMs) *e.g*. cadherins, immunoglobulin-like CAMs (Ig-CAMs), or integrins mediate cell adhesion by homophilic or receptor-ligand interactions^14^. Furthermore, glycans participate in cell adhesion either through direct glycan-glycan interactions, e.g. in some sponges^15^ or bacteria-host cell interactions^16^, or through specific interactions with glycan-binding receptors referred to as lectins^17^. At present, no vesicular prototissues induced by lectin-glycan interactions have been reported. Indeed, the agglutination of vesicles using different lectins and glycolipids were already investigated, but with the aim to study the characteristics of the respective lectins and glycolipids in a simplified model system^18^. In addition, several studies used GUVs to further analyse the receptor binding of e.g. a glycolipid bearing sialyl Lewis^X^ (SLe^X^) to its ligand E-selectin immobilised on planar surfaces or in solution, to investigate the biophysics of cell adhesion (reviewed by Fenz & Sengupta^19^). However, those interactions have not been used to construct larger vesicle networks. Here, we engage multivalent lectins with opposing binding sites to link GUVs presenting carbohydrate moieties on their surface to generate cell-cell junction mimics (hereafter referred to as protocellular junctions). We demonstrate the selectivity of the binding interaction by combining diverse natural, synthetic, and even tailor-made glycoconstructs with different lectins; and the reversibility by ligand competition and lectin inactivation. To further mimic biological conditions, we show that the formation of protocellular junctions can be combined with adhesion to a support through other ligand-receptor interactions; and that lipopolymers can be included to resemble the repulsive character of the glycocalyx of cells. The latter generates the important interplay of specific interaction and unspecific repulsion which both contribute to the complex process of cell adhesion and interaction.

## Results and Discussion

### Selective lectin-glycan interactions induce protocell crosslinking, which results in the formation of prototissues with protocellular junctions

Glycosphingolipids are important components of biological membranes that influence their molecular organisation and architecture^20,21^. Furthermore, they are recognised by carbohydrate-binding proteins and serve as receptors^22^ e.g. for microbial toxins^23^, viruses^24^, and bacteria^25^, and act as mediators of cell adhesion^26^.

**Figure 1:**
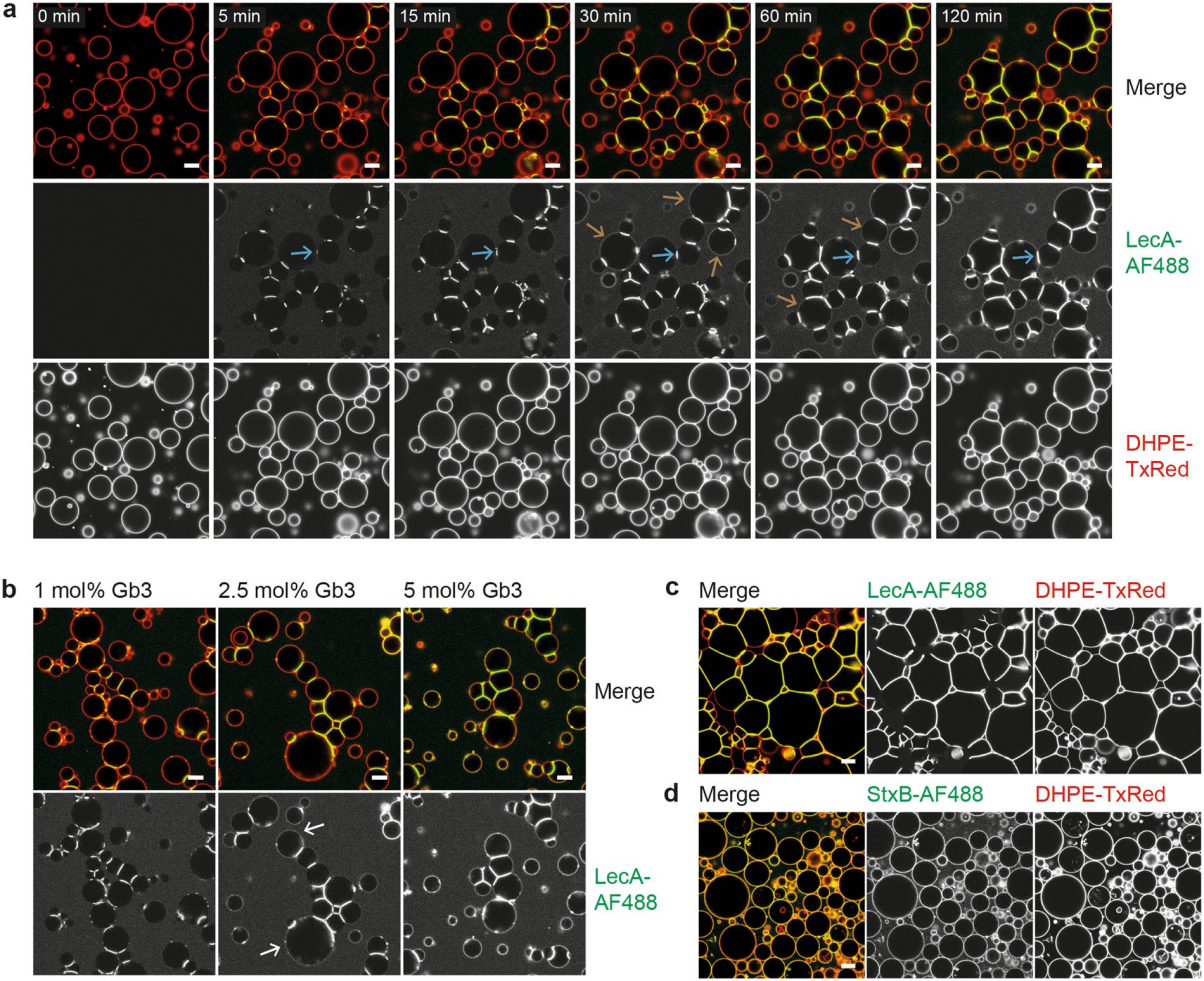
LecA binding to Gb3-functionalised vesicles results in protocell crosslinking and the formation of prototissues. (**a**) Time-lapse of protocell crosslinking after the addition of 100 nM LecA-AF488 (green) to GUVs composed of DOPC:cholesterol:DHPE-TxRed (red) (64.5:30:0.5 mol%, respectively) and 5 mol% Gb3. LecA accumulated within interfaces, which accordingly did grow in size and presented a stronger fluorescence intensity (blue arrows). The lectin signal outside of contact areas increased once the interfaces became saturated (brown arrows at different time points). (**b**) Influence of Gb3 concentration. GUVs composed of 30 mol% cholesterol, 0.5 mol% DHPE-TxRed, and 68.5 mol% DOPC for 1 mol% Gb3, 67 mol% DOPC for 2.5 mol% Gb3, and 64.5 mol% DOPC for 5 mol% Gb3, respectively, were incubated with 100 nM LecA-AF488 for >2 h. The TxRed channel (membrane dye) is depicted in the merge, but not as a separate image, as it does not provide additional information.Vesicles with 1 mol% Gb3 (left panel) showed no lectin accumulation outside of contact areas and less fluorescence within interfaces compared to higher Gb3 concentrations. Functionalisation with 2.5 mol% Gb3 (middle panel) resulted in a more complex situation, where mainly vesicles at the border of clusters showed lectin accumulation outside of interfaces (white arrows). GUVs with 5 mol% Gb3 (right panel) presented a strong overall lectin binding. (**c**,**d**) Vesicles with the same lipid composition as in (**a**) were used. (**c**) Incubation of greater amounts of vesicles with 100 nM LecA-AF488 for >2h resulted in the formation of large prototissues with polygonal geometry. (**d**) Incubation with 100 nM StxB-AF488 (B-subunit of Shiga toxin, green), a homopentameric, Gb3 binding lectin without opposing binding sites, for >2 h resulted in homogenous binding but no formation of protocelluar junctions. Scale bars = 10 µm.

Interestingly, we observed that vesicles functionalised with the glycosphingolipid globotriaosylceramide (Gb3, also known as CD77 or the P^k^ blood group antigen) formed crosslinked vesicle clusters when fluorescently labelled LecA (LecA-AF488, green) was added (Fig. 1a, and Supplementary Movie S1). LecA is a homotetrameric, galactophilic lectin from *Pseudomonas aeruginosa* with one binding pocket per monomer, whose receptor is, amongst others, Gb3^27^. Due to the structural organisation of the LecA tetramer, the binding sites are localised on opposing ends of the protein (Supplementary Fig. S1a), which we suggest is the reason for the ability of LecA to crosslink adjacent Gb3-functionalised vesicles. Moreover, indicated by the increase of fluorescent signal, LecA was strongly accumulating in the interfaces of contacting vesicles (Fig. 1a, blue arrows). The increase of fluorescence especially from unbound LecA over time indicates that the lectin was not fully distributed after addition; yet resuspension could destroy GUVs and result in a fluid flow which would disturb imaging. Hence, the kinetics of lectin binding could be influenced by the diffusion of the lectin within the chamber and result in an increase of lectin binding over a longer time period. However, the background fluorescence of free lectin indicates that LecA was provided in excess and accordingly the kinetics of crosslinking should not be significantly influenced. In contrast to the accumulation of the lectin within interfaces, on individual isolated GUVs the bound lectin showed a homogenous distribution (Supplementary Fig. S2). Considering that (glyco-)lipids freely diffuse in free standing synthetic membranes, we hypothesise that lipids with bound lectins or small lipid-lectin clusters, too, undergo unconstrained diffusion. However, when a lectin with opposing binding sites such as LecA diffuses into the contact area of two adjacent vesicles, the free binding sites that are potentially oriented away from the first membrane can bind to Gb3 molecules of the second GUV. As a consequence, the movement of the lectin is restricted to the interface in order to be in contact with both membranes. Fluorescence recovery after photobleaching (FRAP) experiments indicated that the lectin between the two membranes is still mobile and able to diffuse within the interface area, but with a lower molecular diffusivity compared to LecA bound to the membrane of a single GUV (Supplementary Fig. S3). In addition, the accumulation of lectin results in an enlargement of the interface area so that the GUVs form what we consider protocellular junctions. In the course of time, a fluorescent LecA signal also became apparent outside of contact areas, but with slightly different kinetics for distinct GUVs (Fig. 1a, brown arrows at different time points). We understand this observation as an indication that the growth of the interfaces and accumulation of lectin within the latter reached a saturation level, which we speculate can differ for different vesicles depending on their size, their total amount of Gb3 receptor, and also the number and area sizes of the involved interfaces.

Accordingly, varying concentrations of Gb3 were used to assess the influence of the amount of receptor within protocells (Fig. 1b). We did not observe significant lectin binding outside the contact regions on crosslinked vesicles containing 1 mol% of Gb3, even after incubation times longer than 2 h (Fig. 1b, left panel). This indicates that here the vast majority of lectin-bearing Gb3 molecules accumulated within the contact areas of touching vesicles. Corresponding to the lower Gb3 amount, also overall less fluorescent LecA was binding to these GUVs compared to vesicles containing higher concentrations of receptor. In contrast, when GUVs contained 5 mol% Gb3 (Fig. 1b, right panel) a significant amount of LecA fluorescence was present outside the contact zones. This implies that the concentration of lectin-bearing Gb3 in the interfaces was too high to allow for further membrane-bound lectin to diffuse into the saturated interfaces. The situation was more complex when vesicles contained 2.5 mol% Gb3 (Fig. 1b, middle panel). While the majority of crosslinked liposomes did not present lectin binding outside of interfaces, especially vesicles at the border of crosslinked clusters did show an increase of binding to the latter (Fig. 1b, white arrows). A time-series of LecA binding to GUVs with different Gb3 concentrations is depicted in Supplementary Fig. S4. We understand that this observation is due to the fact that GUVs within a cluster are linked to more than one other vesicle, and that a larger total-interface-area requires more Gb3 to reach saturation. Correspondingly, Supplementary Movie S1 illustrates a time series of a vesicle (pink arrow), which forms an additional contact area after 111 min. Subsequently, the fluorescence signal increased within the new interface, while it decreased outside the contact zone, indicating that the lectin bound to its ligand which could not enter the already saturated interfaces before, accumulated within the newly formed interface.

In general, the growth of the contact area between touching GUVs is also accompanied by a significant vesicle deformation, as LecA-induced interfaces have the tendency to adopt a planar geometry. By employing this effect and by using a greater amount of GUVs, we succeeded to generate not only small vesicle clusters but large prototissues with almost no gaps between liposomes (Fig. 1c). In those tissues, the contact area between GUVs was increased so that the protocells no longer appeared to be spherical but polygonal. We hypothesize that the physical parameters which counteract the interface growth and the accompanied vesicle deformation are a combination of membrane rigidity and membrane tension increase due to volume constraints; although we cannot measure these parameters directly due to the highly complex prototissue architecture. This speculation is mostly in line with findings from a study on adhesion induced GUV deformation^28^. Nevertheless, introducing reasonable differences in osmotic pressure which results in GUV inflation or using a different lipid composition without cholesterol did not significantly affect the GUV deformation process and the formation of protocellular junctions (Supplementary Fig. S5). However, the aim of the present work was not to quantitatively analyse this effect but to use the lectin-mediated crosslinking to generate prototissues for mimicking cellular adhesion.

To affirm that the crosslinking character of LecA results from its topology with opposing binding sites, we used the non-toxic Gb3-binding B-subunit of Shiga toxin (StxB), produced by *Shigella dysenteriae* or enterohaemorrhagic *Escherichia coli* (EHEC) strains, as a control. StxB assembles as a homopentamer with up to three binding sites per monomer and the crystal structure of the complex with Gb3 oligosaccharides demonstrates that all binding pockets are facing towards the same direction^29^ (Supplementary Fig. S1b). Indeed, fluorescently labelled StxB (StxB-AF488, green) did bind to Gb3 containing vesicles, but in contrast to LecA, it did not exhibit any of the described indications of crosslinking, i.e. accumulation within contact areas and elongation of interfaces, even when vesicles were in close proximity (Fig. 1d). In addition, two other lectins with a similar topology to LecA were investigated: a second lectin from *P. aeruginosa*, the tetravalent fucose-binding LecB^30^ (Supplementary Fig. S1c); and the tetravalent legume lectin extracted from *Vicia villiosa* seeds named VVL with a preference for O-linked N-acetylgalactosamine (GalNAc)^31^ (Supplementary Fig. S1d). Indeed, both lectins formed protocellular junctions when added to GUVs containing their respective receptor: the Lewis^a^ (Le^a^) blood group trisaccharide linked via an organic spacer to DOPE (DOPE-Le^a^) for LecB; and a synthetic Cholesterol-Substituted Glycopeptide with a GalNAc-moiety (CSG-peptide) for VVL (Supplementary Fig. S6). The latter is a tailor-made glycopeptide which was previously presented to integrate into GUVs to generate glycodecorated vesicles^32^.

In conclusion, we are able to generate prototissues which resemble the geometry of e.g. epithelial cells within tissues. In addition, the formation of synthetic prototissues using lectin-glycan interactions demonstrates similarities to the cell-cell junction formation through apical adherens junctions during mesenchymal-epithelial transitions (MET) of cell culture studies^33^. In the latter, actin-based cell protrusions generate initial contact sites where cadherins engage in homophilic interactions and form clusters. Synthetic vesicles are not able to form protrusions, but when two vesicles are in close proximity, the formation of protocellular junctions also starts at the point of contact where LecA binds to Gb3 present on both membranes and forms clusters. Following, in cells the cytoskeleton is rearranged driving the contact expansion and the formation of a so called adhesion belt. The increase of contact surface between cells suggests continuous addition of new molecules which accordingly stop to diffuse freely but become immobilised^34^. Accordingly, MET results in the generation of tissues; in contrast to the epithelial-mesenchymal transition (EMT) in which cell-cell adhesions are lost. Strikingly, we are able to mimic this process of contact expansion in a minimal system in which no cytoskeleton is involved, leading to the formation of elongated protocellular junctions mimicking the adhesion belt. Also the timeframe for both the formation of a cadherin adhesion belt and the elongation of protocellular junctions is within a few tens of minutes^34^. Furthermore, the lectin-glycan interactions are highly specific and versatile, as we present a certain variability of lectins which can be used, along further manifold possibilities of glycosphingolipids, glycans synthetically linked to phospholipids, and even tailor-made cholesterylated glycopeptides.

### Generation of more complex protocellular junctions comprising multiple distinct lectin domains

**Figure 2:**
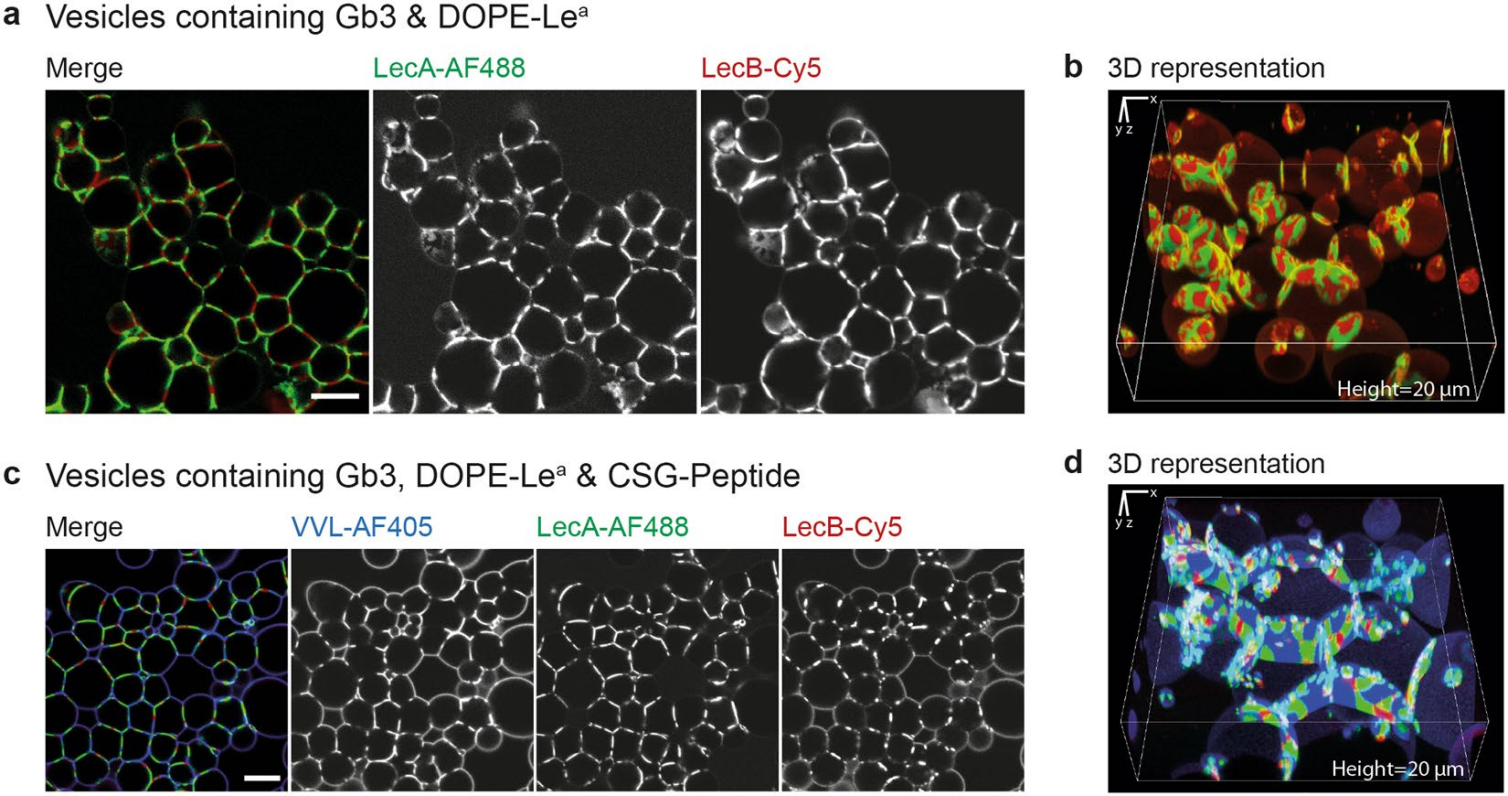
Generation of complex protocellular junctions comprising multiple distinct lectin domains. Different lectins were used in parallel to crosslink liposomes decorated with several glycomodules. (**a**) GUVs composed of 60 mol% DOPC, 30 mol% cholesterol, and each 5 mol% Gb3 and DOPE-Le^a^ were crosslinked with 100 nM of each LecA-AF488 (green) and LecB-Cy5 (dark red) for >2h. LecA and LecB did segregate into distinct domains within contact areas of GUVs but otherwise remained intermixed. (**b**) Representative 3D representation of vesicles with the same lipid composition as in (**a**) illustrating the separate lectin domains within interfaces (**c**) GUVs composed of DOPC (56.7 mol%), cholesterol (28.3 mol%), and Gb3, DOPE-Le^a^, and CSG-peptide (5 mol% each) were crosslinked with 100 nM of each LecA-AF488 (green), LecB-Cy5 (dark red), and VVL-AF405 (blue) for >2h. All three lectins did segregate into distinct domains within interfaces. (**d**) Representative 3D representation depicting the three distinct lectin domains within interfaces of vesicles with the same lipid composition but of slightly bigger size for better visibility as in (**c**). Scale bars = 10 µm.

In the previous experiments we could identify three lectins with opposing binding sites which are able to crosslink adjacent vesicles. The prerequisite that all three lectins display different specificities for galactose, fucose, and GalNAc, respectively, allowed the combination of two or even all three lectins in parallel to generate more complex protocellular junctions. Indeed, when vesicles contained each 5 mol% of Gb3 and DOPE-Le^a^, both LecA-AF488 (green) and LecB-Cy5 (dark red) were involved in the formation of prototissues (Fig. 2a). Interestingly, both lectins segregated into distinct domains within interfaces, while they remained homogenously mixed in the surface area outside of contact zones. The pattern generated by those domains within interfaces can be nicely seen in the 3D representation (Fig. 2b). Accordingly, when the vesicles were decorated with equivalent amounts of the three different glycomodules, Gb3, DOPE-Le^a^, and the CSG-peptide, crosslinking resulted in the segregation of LecA (green), LecB (dark red), and VVL (blue) into distinct domains within interfaces (Fig. 2c). For illustration purposes, slightly larger GUVs are depicted in the 3D representation (Fig. 2d).

It was previously shown that multivalent lectins and bacteria expressing such lectins can induce receptor clustering affecting the membrane organisation^25,35–37^. Furthermore, there is evidence that protein segregation at membrane interfaces^38,39^ is size-dependent and influenced by lateral crowding effects^40^. These aforementioned factors could also affect the lectin segregation within contact areas, as they are all of different size and cluster different receptors. In addition, also the properties of the respective glycomodule, for instance the fatty acid chain length or the length and flexibility of linkers, as well as the length and the branching of the sugars could influence the segregation effect. For StxB it was shown, that also the degree of saturation^23^, as well as the hydroxylation^41^ of different Gb3 species present in distinct cell lines, influence the interaction with its receptor. Besides, it was demonstrated that even though StxB and LecA both bind to Gb3, they segregate into distinct domains on the membrane and can hereafter be trafficked along different intracellular routes^42^.

### The formation of protocellular junctions is reversible

**Figure 3:**
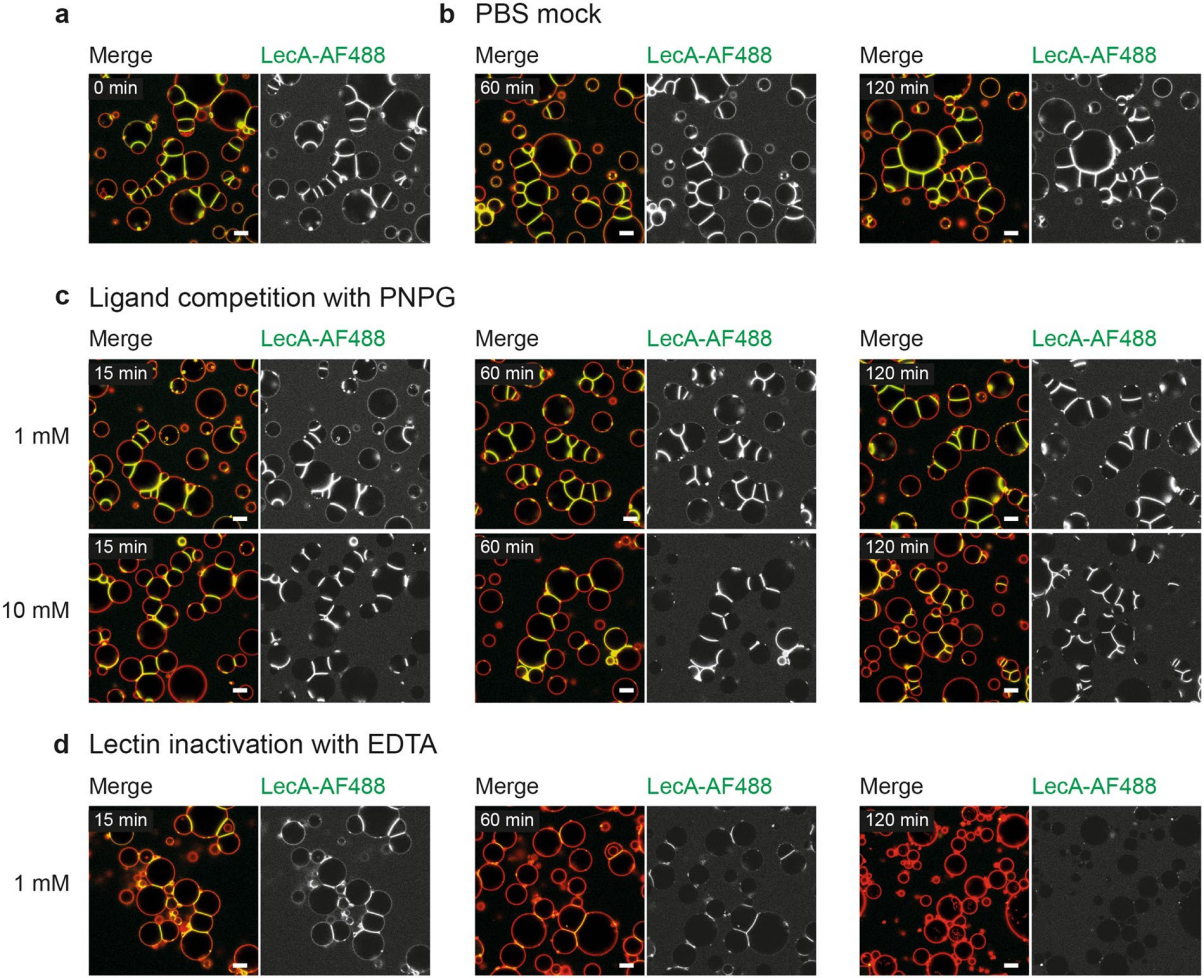
Effect of ligand competition with PNPG, and LecA inactivation with EDTA on lectin binding and crosslinking. Time-series of ligand competition with 4-nitrophenyl-α-D-galactopyranoside (PNPG), or lectin inactivation with ethylenediaminetetraacetic acid (EDTA). Liposomes were composed of DOPC:cholesterol:DHPE-TxRed (red) (64.5:30:0.5 mol%, respectively) and 5 mol% Gb3. The TxRed channel (membrane dye) is depicted in the merge, but not as a separate image, as it does not provide additional information. (**a**) Before treatment, vesicles were crosslinked with 100 nM LecA-AF488 (green) for 120 min. (**b**) PBS mock control to exclude dilution effects. (**c**) Top panel: Addition of 1 mM PNPG remained ineffective also after 2 h of ligand competition. Bottom panel: Addition of 10 mM PNPG resulted in the loss of lectin binding outside of contact areas within the first 15 min. Yet, protocellular junctions remained without significant change even after 120 min. (**d**) Addition of 1 mM EDTA was ineffective for the first 15 min but reversed the formation of protocellular junctions within 120 min. Scale bars = 10 µm.

In contrast to the permanent adhesion for the development of epithelial monolayers, other cellular processes require transient adhesion e.g. leukocytes crossing the vascular endothelium^43^. As the binding of a lectin to its glycan is a reversible interaction influenced by its binding affinity and avidity, we aimed to demonstrate that lectin-glycan-induced protocellular junctions can be reversed to mimic such temporary adhesiveness. The soluble monosaccharide 4-nitrophenyl-α-D-galactopyranoside (PNPG) is a strong affinity ligand for LecA that reduces biofilm formation^44^ as well as the invasiveness^25^ of *P. aeruginosa*. Thus, we generated vesicle clusters by incubating GUVs with LecA for 120 min (Fig. 3a) and used different concentrations of PNPG to compete with the binding of LecA to Gb3 (Fig. 3c) in comparison to a PBS mock control (Fig. 3b). While the addition of 1 mM PNPG remained ineffective even after 120 min (Fig. 3c, top panel), the addition of 10 mM PNPG resulted in the loss of LecA-AF488 binding outside of interfaces within the first 15 min. Furthermore, also the amount of fluorescence within contact zones decreased slightly after 120 min of incubation (Fig. 3c, bottom panel). However, elongated contact areas with high lectin concentration indicating protocellular junctions remained.

Another approach to reverse LecA binding to its receptor is to utilise the calcium chelator ethylenediaminetetraacetic acid (EDTA). LecA possesses a calcium and magnesium dependency for binding, thus the removal of calcium results in inactivation of the lectin^45^. Subsequently, crosslinked vesicles were treated with 1 mM EDTA (Fig. 3d). 15 min after addition, no major difference between the EDTA-treated and the PBS mock-treated samples was detectable. However, within 60 min of treatment, the lectin signal outside the interfaces was completely lost and the fluorescence within the contact zones also decreased significantly. The most impressive result was obtained after 120 min of treatment, where vesicles returned to the geometry of spheres and no protocellular junctions remained.

With these experiments we demonstrate that both ligand competition as well as lectin inactivation can reverse lectin binding. However, only inactivation of the calcium-dependent lectin with EDTA can efficiently reverse the formation of protocellular junctions. One explanation is that ligand competition is governed by the kinetics of ligand dissociation and association. Thus, with higher concentrations of the competitor, the binding equation is shifted towards PNPG compared to Gb3 outside of contact regions. Yet, within interfaces the local concentration of Gb3 is supposedly highly enriched as indicated by the previous experiments. Hence, the affinity towards another Gb3 molecule on the membrane should be higher compared to free PNPG, since the dissociation of one ligand should not change the orientation towards the membrane introduced by the binding of the other three ligands. In addition, a diminished diffusion of PNPG into the interfaces might have a further negative impact, but this question cannot be addressed from our data. Consequently, we propose that the ligand competition is locally less effective within interfaces and this effect will remain mostly independent of the concentration of the competitor in solution.

The use of EDTA renders different results. Calcium ions act as a bridge between the protein and the carbohydrate which is a prerequisite of binding. Accordingly, EDTA does not compete for glycan binding but reversibly inactivates LecA as previously shown for other lectins^46–48^. Hence, higher Gb3 concentrations within interfaces cannot influence the lectin inactivation. Furthermore, EDTA forms a very stable chelating complex so that it can sequester one calcium ion at a time and thereby release the lectin. Again, our data allow no conclusion on the diffusion efficiency of EDTA into interfaces which could influence the kinetics of the disassembly process.

Interestingly, also the cell adhesion molecule family of cadherins, which extensively contributes to the dynamics of cell-cell contact formation, exhibits a dependence on extracellular calcium. Chelation of the latter resulted in a sudden, but partial loss of homophilic interactions^49^. Furthermore, also the molecule family of selectins, which meditate adhesive interactions between leukocytes and the endothelium during inflammation, is calcium-dependent^50^.

### Combined adhesion between protocells and surfaces

**Figure 4:**
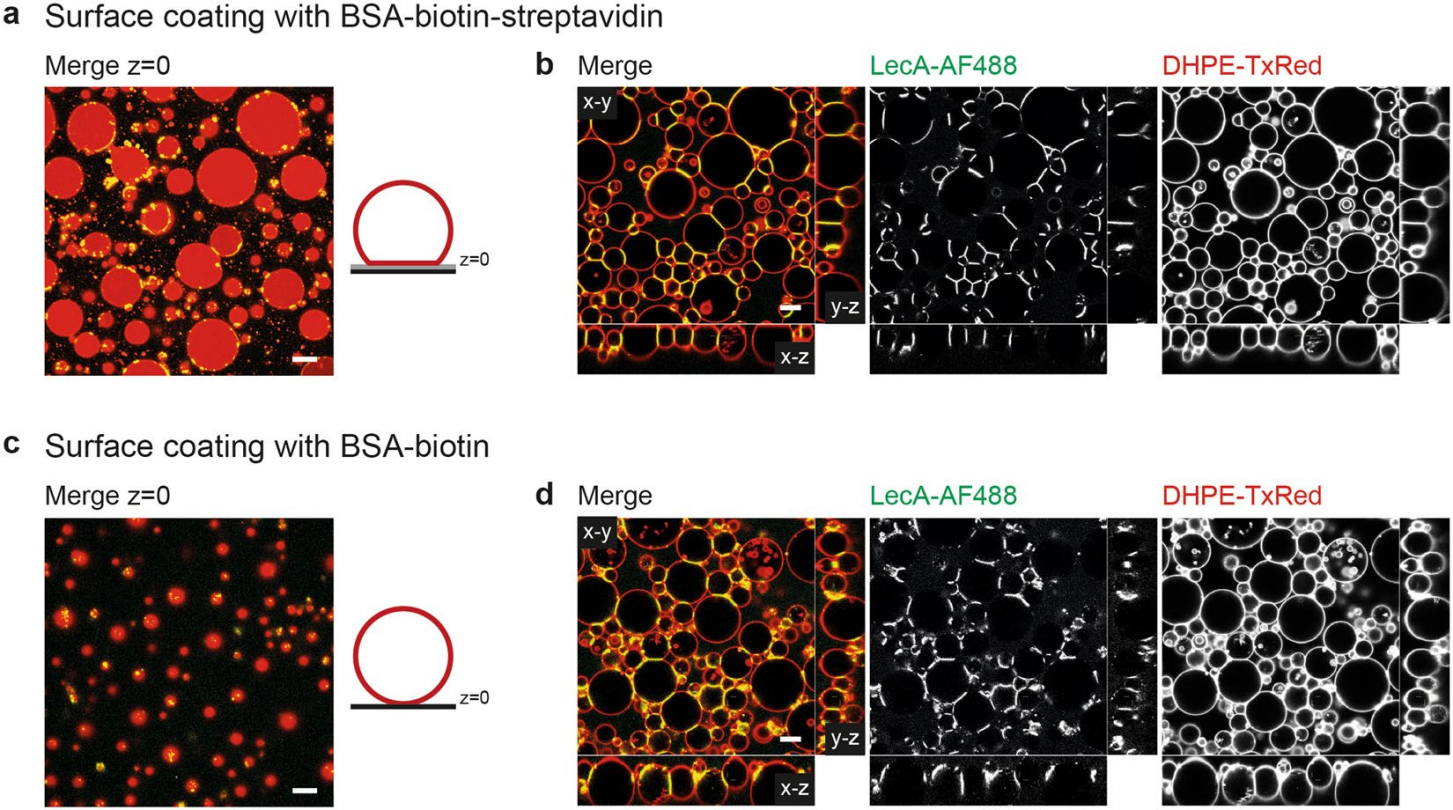
Formation of protocellular junctions combined with or without biotin-streptavidin-mediated attachment to the support. GUVs composed of 64 mol% DOPC, 30 mol% cholesterol, 0.5 mol% DHPE-TxRed (red), 5 mol% Gb3, and 0.5 mol% DOPE-biotin were sedimented and given time to spread for 30 min prior to the addition of 100 nM LecA-AF488 (green) for >2h. (**a,b**) Chambers were coated with BSA-biotin followed by streptavidin and washing to remove unbound residues. (**a**) GUVs formed adhesion patches on the surface (z = 0). For better understanding a scheme illustrates the vesicle deformation on the streptavidin-coated (grey) support (black). (**b**) Representative x-y, x-z, and y-z cross-section of crosslinked vesicles in combination with adhesion to the support. (**c,d**) Chambers were coated with BSA-biotin as a control, followed by washing to remove unbound residues (**c**) GUVs did not interact with the surface and no adhesion patch was formed. For better understanding a scheme illustrates the vesicle on the support (black). (**d**) Representative x-y, x-z, and y-z cross-section of crosslinked vesicles. Scale bars = 10 µm.

To advance the mimicry of more sophisticated prototissues, we aimed to crosslink vesicles in combination with adhesion to a support. A simple system used by several groups is the biotin-avidin/streptavidin ligand-receptor pair generating strong adhesion bonds^51^. Accordingly, the incubation chambers were coated with BSA-biotin followed by a washing step with PBS to remove any unbound remains and incubation with streptavidin to bind to the immobilised biotin molecules. Addition of vesicles functionalised with a small amount of biotin linked via an organic spacer to DOPE (DOPE-biotin) resulted in the typical stages of spreading, as reviewed before^19^, and finally in an adhesion patch (Fig. 4a), which was not observed when the support was only coated with BSA-biotin (Fig. 4c). 100 nM LecA-AF488 was added after the vesicle adhesion was completed to favor x-y crosslinking and not the generation of vesicle piles. Yet, in order to enable crosslinking after adhesion to the support, dense vesicle arrangements were required so that vesicles were in close proximity. By doing so it could not be avoided that some, especially small vesicles, did not sediment to the ground but were staying on top of other liposomes. In contrast to living cells, these vesicles were not able to actively migrate to the ground.

Addition of LecA resulted in the formation of protocellular junctions as presented before. The flat adhesion patch combined with the planar interfaces enhanced the resemblance of epithelial tissues in the x-z and y-z cross-section view (Fig. 4b). In contrast, without the adhesion to streptavidin, GUVs only exhibited loose contacts with the support and appeared rather uneven (Fig. 4d). Furthermore, attachment to the functionalised support enabled the prototissues to withstand the fluid flow generated by resuspension of one third of the chamber volume in 63% of all cases compared to unattached tissues which were always flushed away (38 replicates, Supplementary Fig. S7). The latter effect was also observed by others when single vesicles were attached to surfaces^52^. In nature, cells make close contact to the extracellular matrix (ECM) for exactly the same reason. The ECM is a collagen scaffold with different adhesive glycoproteins, for instance laminin, tenascin, and proteoglycans which provides structural support to tissues^53,54^.

### Influence of steric coating with PEG-modified lipids on the formation of protocellular junctions

**Figure 5:**
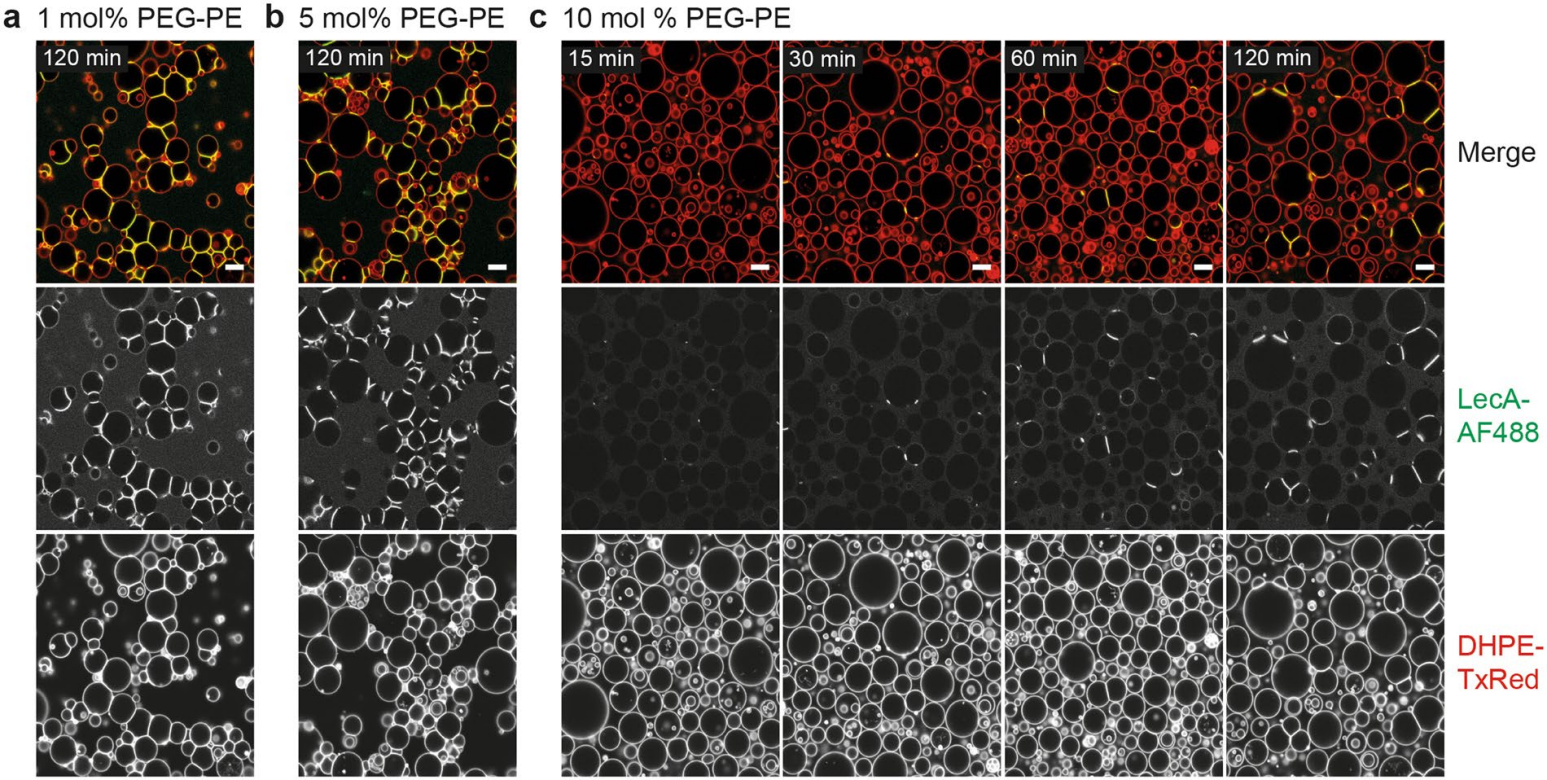
Influence of PEG-modified lipids on lectin binding and the formation of protocellular junctions. GUVs with differing amounts of the lipopolymer 18:0 PEG2000 PE referred to as PEG-PE were crosslinked with 100 nM LecA-AF488 (green) for different time periods. (**a**) GUVs were composed of DOPC:cholesterol:DHPE-TxRed:Gb3 (62.4:31.1:0.5:5 mol%, respectively) and 1 mol% PEG-PE. Protocellular junctions were formed and lectin binding outside of interfaces was observed. (**b**) GUVs with a lipid composition of 59.7 mol% DOPC, 29.8 mol% cholesterol, 0.5 mol% DHPE-TxRed, 5 mol% Gb3, and 5 mol% PEG- PE formed protocellular junctions despite the higher concentration of lipopolymer, but lectin binding outside of contact areas was significantly decreased. (**c**) GUVs were composed of 56.4 mol% DOPC, 28.1 mol% cholesterol, 0.5 mol% DHPE-TxRed, 5 mol% Gb3, and 10 mol% PEG-PE. Only a minority of vesicles showed lectin binding and formation of protocellular junctions although they were in contact, but with increasing quantity after 60–120 min, respectively. Scale bars = 10 µm.

The plentifulness of glycolipids, glycoproteins, and proteoglycans all constitute to the outermost layer of every living cell referred to as glycocalyx^55,56^. Accordingly, cell adhesion is a complex process induced by specific ligand-receptor interactions which compete with unspecific repulsion forces generated by the glycocalyx^57–60^, which can be mimicked by inserting lipopolymers^38,51^. Hence, we incorporated differing amounts (1, 5, and 10 mo%) of 1,2-distearoyl-*sn*-glycero-3-phosphoethanolamine-N-[methoxy(polyethylene glycol)-2000] (18:0 PEG2000 PE hereafter referred to as PEG-PE) into vesicles containing 5 mol% Gb3 to investigate the effect on the formation of lectin-mediated protocellular junctions. For vesicles containing either 1 mol% or 5 mol% PEG-PE, no evident changes in the ability to crosslink were observed (Fig. 5a,b). We propose, that the lectin-glycan interaction is strong enough to overcome the repulsion and PEG-PE is expelled from the adhesion site. Only the amount of lectin bound outside of interfaces was higher when the GUVs were doped with only 1 mol% compared to 5 mol% lipopolymer. A time-series of all concentrations is depicted in Supplementary Fig. S8.

In contrast, vesicles doped with 10 mol% of the lipopolymer were engaged in strikingly less events of crosslinking, although the GUVs were in close proximity (Fig. 5c). Besides, not only interfaces were affected, also a minority of isolated vesicles presented homogenous lectin binding, yet with increasing quantity over time. The latter might indicate a slower kinetics of both binding and crosslinking. Also, when interfaces were formed they revealed the same accumulation of lectin, indicated by an increase of fluorescence, and elongation as protocellular junctions without PEG hindrance. Accordingly, one can assume that the ligand Gb3 is hidden within the layer of large lipopolymers. Hence, the expulsion of the “repeller” from the origin of binding is required, which might considerably decrease the kinetics of the process.

From these experiments we conclude that the binding strength of LecA to Gb3 is strong enough to overcome the repulsion generated by 1–5 mol% of PEG-PE, a concentration often used to model the forces of the glycocalyx^51,61^. Only for as high concentrations as 10 mol% lipopolymer, lectin binding and accordingly the formation of protocellular junctions is indeed influenced by the ligand shielding effect for the majority of vesicles. Furthermore, although referring to a lectin-glycan interaction, it is indeed a glycan-lectin-glycan composition. Correspondingly, while the lectin has to overcome the “repeller” twice instead of itself being shielded by the large head groups of the lipopolymer, a lectin bound to its glycolipid changes the size proportion. Further studies in which vesicles could be directly doped with a membrane standing lectin might generate another valuable system. At last, the incorporation of cadherins or other glycoproteins would give an even more realistic protocellular mimic, however, even though there have been several advances in the formation of proteoliposomes, those techniques render other limitations and are of less variability as reviewed by Lagny & Bassereau^62^.

## Conclusion

Adhesion and tissue formation are very important principles of life. Even though there have been recent advances in the investigation of *e.g*. the mechanics of individual protocells to soluble or immobilised ligands on a support, or the formation of vesicle aggregates by electrostatic attraction in the context of primitive cell communities, no synthetic vesicle model of prototissues using lectin-glycan interactions exists. Here we present that lectins with opposing binding sites which recognise natural and synthetic glycoconjugates can be used to generate protocells linked by protocellular junctions in order to form durable prototissues. We further increase the biological relevance by combining the crosslinking with adhesion to a functionalised support, and by including lipopolymers to mimic the interplay of specific attraction and unspecific repulsion. Accordingly, we propose that the presented protocell/prototissue study provides a novel input towards the construction of the fundamental processes of life.

## Methods

### Materials

1,2-dioleoyl-*sn*-glycero-3-phosphocholine (DOPC), 1,2-distearoyl-*sn*-glycero-3-phosphoethanolamine-N-[methoxy(polyethylene glycol)-2000] (referred to as 18:0 PEG2000 PE), and cholesterol were purchased from Avanti Polar Lipids; Bodipy FL C5-HPC and Texas Red 1,2-dihexadecanoyl-*sn*-glycero-3-phosphaethanolamine (DHPE-TxRed) from Life Technologies; and purified porcine Gb3 from Matreya. FSL-Le^a^(tri) referred to as DOPE-Le^a^(tri), FSL-biotin referred to as DOPE-biotin, 4-nitrophenyl-α-D-galactopyranoside (PNPG), and ethylenediaminetetraacetic acid (EDTA) were obtained from Sigma-Aldrich. The Cholesterol-Substituted Glycopeptide with a GalNAc-moiety (CSG-peptide)^32^ was received from Prof. Klas Ola Blixt (University of Copenhagen). Recombinant LecA and LecB were produced from *Escherichia coli* following previously published procedures^27,63^. VVL was purchased from Vector Laboratories; recombinant Shiga toxin 1 B-subunit (StxB) from Sigma-Aldrich.

### Lectin labelling

Lectins were labelled with Alexa Fluor 405 NHS Ester, Alexa Fluor 488 NHS Ester (Molecular Probes) or Cy5 NHS Ester (GE Healthcare) according to the manufacturer’s protocol for amine-reactive probes. Free dye was removed using Zeba Spin desalting columns with 7K MWCO (Thermo Fisher Scientific).

### GUV preparation

GUVs were prepared using the electroformation technique as previously described by Madl *et al*.^64^. In brief, lipids dissolved in chloroform to a total concentration of 0.5 mg ml^−1^ were spread on indium-tin oxide (ITO)-coated slides. After solvent evaporation, electroformation was carried out at room temperature for 3 h in ~265 mOsm L^−1^ sucrose by applying an alternating electric field with a field strength of 1 V/mm. If not indicated otherwise, the lipid preparations contained 30 mol% cholesterol, 0.5 mol% DHPE-TxRed, indicated concentrations of 1,2-dioleoyl-sn-glycero-3- phosphocholine (DOPC), and indicated concentrations of the glycosphingolipid Gb3, the blood group sugar Lewis^a^ (Le^a^) or biotin linked via an organic spacer to DOPE (DOPE-Le^a^; DOPE-biotin), a synthetic Cholesterol-Substituted Glycopeptide with a GalNAc-moiety (CSG-peptide), or 18:0 PEG2000 PE (referred to as PEG-PE). Primarily in lipid mixes containing more than one functionalised lipid, a cholesterol:DOPC ratio of 2:1 was used to circumvent too low concentrations of DOPC.

### Observation of lectin binding and vesicle aggregation

For the observation of lectin binding and vesicle aggregation, home-built chambers were used as described in Madl *et al*.^64^. In short, 8 × 8 mm cloning cylinders were glued to coverslips to enable the possibility to add LecA or other compounds. The chambers were coated with β-Casein (Sigma-Aldrich) to prevent unspecific adhesion and vesicle rupture. GUVs sedimented in such chambers filled with PBS (matched osmolarity to sucrose) due to their higher density. After sedimentation, crosslinking mainly occurred in the x-y and not z-direction. The lectins were added to the GUVs in an equivalent volume of PBS to the volume of PBS and GUVs in sucrose to constitute an evenly distributed intermixture of a final concentration of 100 nM lectin. Only in Fig. 3, LecA was added to PBS to yield a concentration of 100 nM before the addition of the GUVs, as the inhibitors were added subsequently.

### Streptavidin coating and vesicle adhesion

To generate adhesion to the surface, the chamber was first coated with 0.1 mg/ml BSA-biotin for 2 h at room temperature, followed by coating with 0.1 mg/ml streptavidin for 30 min, and three washing steps with PBS. For the control, chambers were coated only with 0.1 mg/ml BSA/biotin for 2.5 h followed by similar washing.

### PNPG competition and EDTA inactivation

After vesicles were crosslinked with 100 nM lectin, an equivalent volume of the competitors in PBS to the volume of GUVs and lectin in PBS was added to enable an equal distribution. For a mock control the same volume of PBS was added to exclude dilution effects.

### Microscopic imaging

GUVs were imaged on a confocal microscope (Nikon Eclipse Ti-E inverted microscope equipped with a Nikon A1R confocal laser scanning system, 60x oil immersion objective, NA = 1.49, 4 laser lines: 405 nm, 488 nm, 561 nm, 640 nm; Nikon Instruments). The software NIS-elements (Nikon) was used for image acquisition and analysis.

### Data availability

The datasets generated during and/or analysed during the current study are available from the corresponding author on reasonable request.

## Electronic supplementary material

Supplementary Information

LecA crosslinking of vesicles containing Gb3, related to Fig. 1a

## References

[1] Schwille P (2011). Engineering in a Tinkerer’s World..

[2] Benner SA, Sismour AM (2005). Synthetic biology. Nat. Rev. Genet..

[3] Szostak JW (2001). Synthesizing life. Nature.

[4] Luisi PL (2002). Toward the engineering of minimal living cells. Anat. Rec..

[5] Beales PA, Kyle Vanderlick T (2007). Specific binding of different vesicle populations by the hybridization of membrane-anchored DNA. J. Phys. Chem. A.

[6] Beales PA, Nam J, Vanderlick TK (2011). Specific adhesion between DNA-functionalized ‘Janus’ vesicles: size-limited clusters. Soft Matter.

[7] Beales Pa, Vanderlick TK (2014). Application of nucleic acid-lipid conjugates for the programmable organisation of liposomal modules. Adv. Colloid Interface Sci..

[8] Chiruvolu S (1994). Higher order self-assembly of vesicles by site-specific binding. Science (80-.)..

[9] Walker SA, Kennedy MT, Zasadzinski JA (1997). Encapsulation of bilayer vesicles by self-assembly. Nature.

[10] Menger FM, Seredyuk VA, Yaroslavov AA (2002). Adhesive and anti-adhesive agents in giant vesicles. Angew. Chemie - Int. Ed..

[11] Robert É (2015). Mimicking and Understanding the Agglutination Effect of the Antimicrobial Peptide Thanatin Using Model Phospholipid Vesicles. Biochemistry.

[12] Carrara P, Stano P, Luisi PL (2012). Giant Vesicles ‘Colonies’: A Model for Primitive Cell Communities. ChemBioChem.

[13] Souza, T. *et al*. Vesicle aggregates as a model for primitive cellular assemblies. *Phys. Chem. Chem. Phys*, **19**, 20082–20092 (2017). 10.1039/c7cp03751a 28726904

[14] Cavallaro U, Dejana E (2011). Adhesion molecule signalling: not always a sticky business. Nat. Rev. Mol. Cell Biol..

[15] Bucior I, Scheuring S, Engel A, Burger MM (2004). Carbohydrate–carbohydrate interaction provides adhesion force and specificity for cellular recognition. J. Cell Biol..

[16] Day CJ (2015). Glycan:glycan interactions: High affinity biomolecular interactions that can mediate binding of pathogenic bacteria to host cells. Proc. Natl. Acad. Sci..

[17] Taylor ME, Drickamer K (2007). Paradigms for glycan-binding receptors in cell adhesion. Current Opinion in Cell Biology.

[18] Hoekstra D, Düzgünes N, Wilschut J (1985). Agglutination and Fusion of Globoside GL-4 Containing Phospholipid Vesicles Mediated by Lectins and Calcium Ionst. Biochemistry.

[19] Fenz SF, Sengupta K (2012). Giant vesicles as cell models. Integr. Biol..

[20] Simons K, Ikonen E (1997). Functional rafts in cell membranes. Nature.

[21] Lingwood, C. A. Glycosphingolipid functions. *Cold Spring Harb. Perspect. Biol*. **3**, a004788 (2011). 10.1101/cshperspect.a004788 PMC3119914 21555406

[22] Aigal S, Claudinon J, Römer W (2014). Plasma membrane reorganization: A glycolipid gateway for microbes. Biochim. Biophys. Acta.

[23] Römer W (2007). Shiga toxin induces tubular membrane invaginations for its uptake into cells. Nature.

[24] Ewers H (2010). GM1 structure determines SV40-induced membrane invagination and infection. Nat. Cell Biol..

[25] Eierhoff T (2014). A lipid zipper triggers bacterial invasion. Proc. Natl. Acad. Sci. USA.

[26] Regina Todeschini A, Hakomori SI (2008). Functional role of glycosphingolipids and gangliosides in control of cell adhesion, motility, and growth, through glycosynaptic microdomains. Biochim. Biophys. Acta - Gen. Subj..

[27] Blanchard B (2008). Structural basis of the preferential binding for globo-series glycosphingolipids displayed by Pseudomonas aeruginosa lectin I. J. Mol. Biol..

[28] Steinkühler J, Agudo-Canalejo J, Lipowsky R, Dimova R (2016). Modulating Vesicle Adhesion by Electric Fields. Biophys. J..

[29] Ling H (1998). Structure of the Shiga-like toxin I B-pentamer complexed with an analogue of its receptor Gb3. Biochemistry.

[30] Mitchell E (2002). Structural basis for oligosaccharide-mediated adhesion of Pseudomonas aeruginosa in the lungs of cystic fibrosis patients. Nat. Struct. Biol..

[31] Babino A (2003). The crystal structure of a plant lectin in complex with the Tn antigen. FEBS Lett..

[32] Stuhr-Hansen N (2016). Synthesis of Cholesterol-Substituted Glycopeptides for Tailor-Made Glycocalyxification of Artificial Membrane Systems. ChemBioChem.

[33] Cavey M, Lecuit T (2009). Molecular bases of cell-cell junctions stability and dynamics. Cold Spring Harb. Perspect. Biol..

[34] Adams CL, Chen YT, Smith SJ, Nelson WJ (1998). Mechanisms of epithelial cell-cell adhesion and cell compaction revealed by high-resolution tracking of E-cadherin-green fluorescent protein. J. Cell Biol..

[35] Windschiegl B (2009). Lipid reorganization induced by Shiga toxin clustering on planar membranes. PLoS One.

[36] Schütte OM (2014). Influence of Gb3 glycosphingolipids differing in their fatty acid chain on the phase behaviour of solid supported membranes: chemical syntheses and impact of Shiga toxin binding. Chem. Sci..

[37] Schütte OM (2015). 2-Hydroxy Fatty Acid Enantiomers of Gb3 Impact Shiga Toxin Binding and Membrane Organization. Biophys. J..

[38] Limozin L, Sengupta K (2007). Modulation of vesicle adhesion and spreading kinetics by hyaluronan cushions. Biophys. J..

[39] Weikl TR, Asfaw M, Krobath H, Różycki B, Lipowsky R (2009). Adhesion of membranes via receptor–ligand complexes: Domain formation, binding cooperativity, and active processes. Soft Matter.

[40] Schmid EM (2016). Size-dependent protein segregation at membrane interfaces. Nat. Phys..

[41] Römer W (2010). Actin dynamics drive membrane reorganization and scission in clathrin-independent endocytosis. Cell.

[42] Müller, S. K. *et al*. Gb3-binding lectins as potential carriers for transcellular drug delivery. *Expert Opin. Drug Deliv.* **14**, 141–153 (2017). 10.1080/17425247.2017.1266327 27935765

[43] Vestweber, D. How leukocytes cross the vascular endothelium. *Nature Reviews Immunology* **15,** 692–704 (2015). 10.1038/nri3908 26471775

[44] Diggle SP (2006). The galactophilic lectin, LecA, contributes to biofilm development in Pseudomonas aeruginosa. Environ. Microbiol..

[45] Imberty A, Wimmerová M, Mitchell EP, Gilboa-Garber N (2004). Structures of the lectins from Pseudomonas aeruginosa: insights into the molecular basis for host glycan recognition. Microbes Infect..

[46] Konozy EH (2003). Isolation, purification, and physicochemical characterization of a d-galactose-binding lectin from seeds of Erythrina speciosa. Arch. Biochem. Biophys..

[47] Duk M, Lisowska E (1984). Effect of pH on the binding of Vicia graminea lectin to erythrocytes: Dependence on the chemical character of red‐cell receptors. Eur. J. Biochem..

[48] Roberts DD, Goldstein IJ (1984). Effect of carbohydrate and metal ion inding on the reactivity of the essential thiol groups of lima beam lectin. J. Biol. Chem..

[49] Kim SA, Tai C-Y, Mok L-P, Mosser EA, Schuman EM (2011). Calcium-dependent dynamics of cadherin interactions at cell-cell junctions. Proc. Natl. Acad. Sci. USA.

[50] Aplin AE, Howe A, Alahari SK, Juliano RL (1998). Signal transduction and signal modulation by cell adhesion receptors: the role of integrins, cadherins, immunoglobulin-cell adhesion molecules, and selectins. Pharmacol. Rev..

[51] Albersdörfer A, Feder T, Sackmann E (1997). Adhesion-induced domain formation by interplay of long-range repulsion and short-range attraction force: a model membrane study. Biophys. J..

[52] Wattenbarger MR, Graves DJ, Lauffenburger DA (1990). Specific adhesion of glycophorin liposomes to a lectin surface in shear flow. Biophys. J..

[53] Bosman FT, Stamenkovic I (2003). Functional structure and composition of the extracellular matrix. J. Pathol..

[54] Hynes RO (2009). The Extracellular Matrix: Not Just Pretty Fibrils. Science (80-.)..

[55] Bennett HS (1963). Morphological aspects of extracellular polysaccharides. J. Histochem. Cytochem..

[56] Mager MD, LaPointe V, Stevens MM (2011). Exploring and exploiting chemistry at the cell surface. Nat. Chem..

[57] Bell GI, Dembo M, Bongrand P (1984). Cell adhesion. Competition between nonspecific repulsion and specific bonding. Biophys. J..

[58] Foa C, Soler M, Benoliel A-M, Bongrand P (1996). Steric stabilization and cell adhesion. J. Mater. Sci. Mater. Med..

[59] Sackmann E, Bruinsma RF (2002). Cell adhesion as wetting transition?. ChemPhysChem.

[60] Sackmann E, Goennenwein S (2006). Cell Adhesion as Dynamic Interplay of Lock-and-Key, Generic and Elastic Forces. Prog. Theor. Phys. Suppl..

[61] Kloboucek A, Behrisch A, Faix J, Sackmann E (1999). Adhesion-Induced Receptor Segregation and Adhesion Plaque Formation: A Model Membrane Study. Biophys. J..

[62] Lagny, T. J. & Bassereau, P. Bioinspired membrane-based systems for a physical approach of cell organization and dynamics: usefulness and limitations. *Interface Focus* **5**, 20150038 (2015). 10.1098/rsfs.2015.0038 PMC4590427 26464792

[63] Kostlánová N (2005). The fucose-binding lectin from Ralstonia solanacearum. A new type of beta-propeller architecture formed by oligomerization and interacting with fucoside, fucosyllactose, and plant xyloglucan. J. Biol. Chem..

[64] Madl, J., Villringer, S. & Römer, W. in *Chemical and Synthetic Approaches in Membrane Biology* (ed. Shukla, A.) 17–23 (Humana Press, 2017).

