# Supplementary material for: Lectin-mediated protocell crosslinking to mimic cell-cell junctions and adhesion
Source: Sci Rep. 2018 Jan 31;8:1932. doi: 10.1038/s41598-018-20230-6 (PMC5792463; doi:10.1038/s41598-018-20230-6)
Supplement: Supplementary file 1 — Supplementary Information [file 41598_2018_20230_MOESM1_ESM.pdf]

Supplementary Information

Lectin-mediated protocell crosslinking to mimic cell-cell junctions and adhesion

Sarah Villringer[1,2], Josef Madl[1,2,*], Taras Sych[1,2,3,4], Christina Manner[2,§], Anne Imberty[5], and Winfried Römer [1,2,3,*]

1 Faculty of Biology, Albert-Ludwigs-University Freiburg, Schänzlestraße 1, 79104 Freiburg, Germany

2 Bioss - Centre for Biological Signalling Studies, Albert-Ludwigs-University Freiburg, Schänzlestraße 18, 79104 Freiburg, Germany

3 Freiburg Center for Interactive Materials and Bioinspired Technology (FIT), Albert-Ludwigs-University Freiburg, Georges-Köhler-Allee 105, 79110 Freiburg, Germany

4 Laboratoire de Biophotonique et Pharmacologie, UMR 7213 CNRS, Faculté de Pharmacie, Université de Strasbourg, 67401 Illkirch Cedex, France

5 CERMAV UPR5301 CNRS, Université Grenoble Alpes, 38041 Grenoble, France

§ Current address: Focal Area of Infection Biology, Biozentrum, University of Basel, 4056 Basel, Switzerland

Supplementary Methods

FRAP experiments

Fluorescence recovery after photobleaching (FRAP) experiments were carried out by bleaching a rectangular region of interest (ROI) for 1 sec with 100% laser power of the 488 nm laser. Imaging was performed at 1% laser power. Three images were recorded before the bleaching pulse. To monitor the fast recovery directly after bleaching 50 images were taken with a scan speed of 0.07 sec/frame followed by 50 images with a 1 sec interval.

In order to depict the recovery curve, the mean fluorescence intensities (F) within the ROI were normalized to the mean fluorescence intensity of the saturation level (F_{sat}) and plotted over time.

Osmolartiy studies

To assess the influence of the osmolarity on the formation of protocellular junctions, GUVs were electroformated in sucrose of ~290 mOsm. To increase the osmotic mismatch of the hyperosmotic vesicles compared to the aqueous surrounding, PBS was diluted with water to ~230 mOsm.

Supplementary Figures

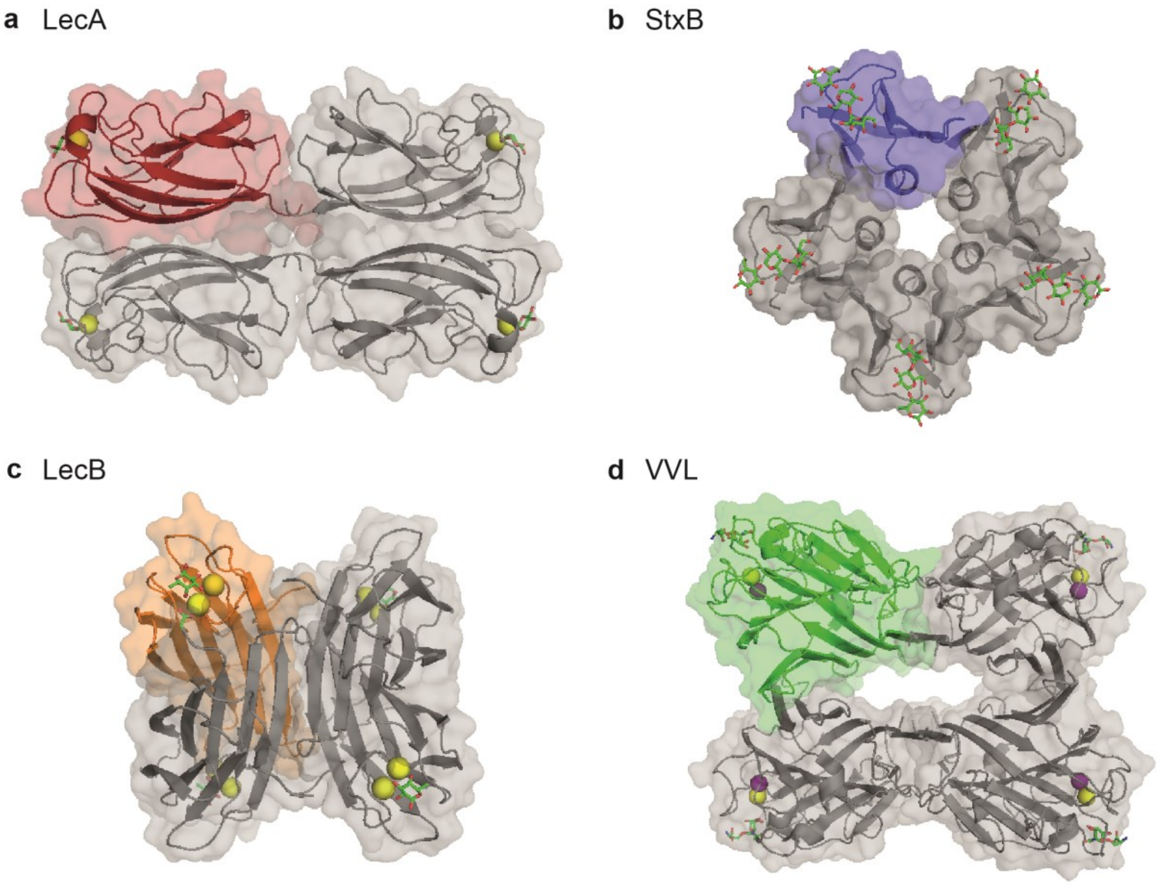

Supplementary Figure S1: Lectin crystal structures

Crystal structures of the respective lectins with stick representation of carbohydrates and cpk representation of calcium (yellow) and manganese (mangenta) ions. Images were generated using the indicated pdb coordinates from the RCSB Protein Data Bank with PyMOL (**a**) Pseudomonas aeruginosa lectin LecA complexed with galactose; pdb 1OKO; from Cioci et al., (2003) [59]. (**b**) Non-toxic B-subunit of Shiga toxin (StxB) complexed with Gb3 trisaccharide starfish; pdb 1QNU; from Kitov et al., (2000) [60]. (**c**) Pseudomonas aeruginosa lectin LecB complexed with with fucose; pdb 1GZT; from Mitchell et al., (2002) [29]. (**d**) Vicia villosa lectin VVL in complex with the Tn antigen; pdb 1N47; from Babino et al., (2003) [30].

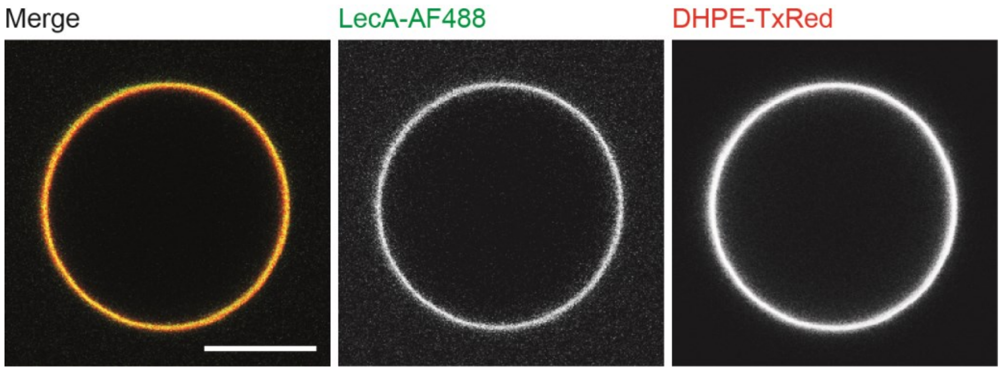

Supplementary Figure S2: Homogeneous LecA binding to isolated vesicles

Homogenous binding of 100 nM LecA-AF488 to isolated GUVs composed of DOPC:cholesterol:DHPE-TxRed (red) (64.5:30:0.5 mol%, respectively) and 5 mol% Gb3.

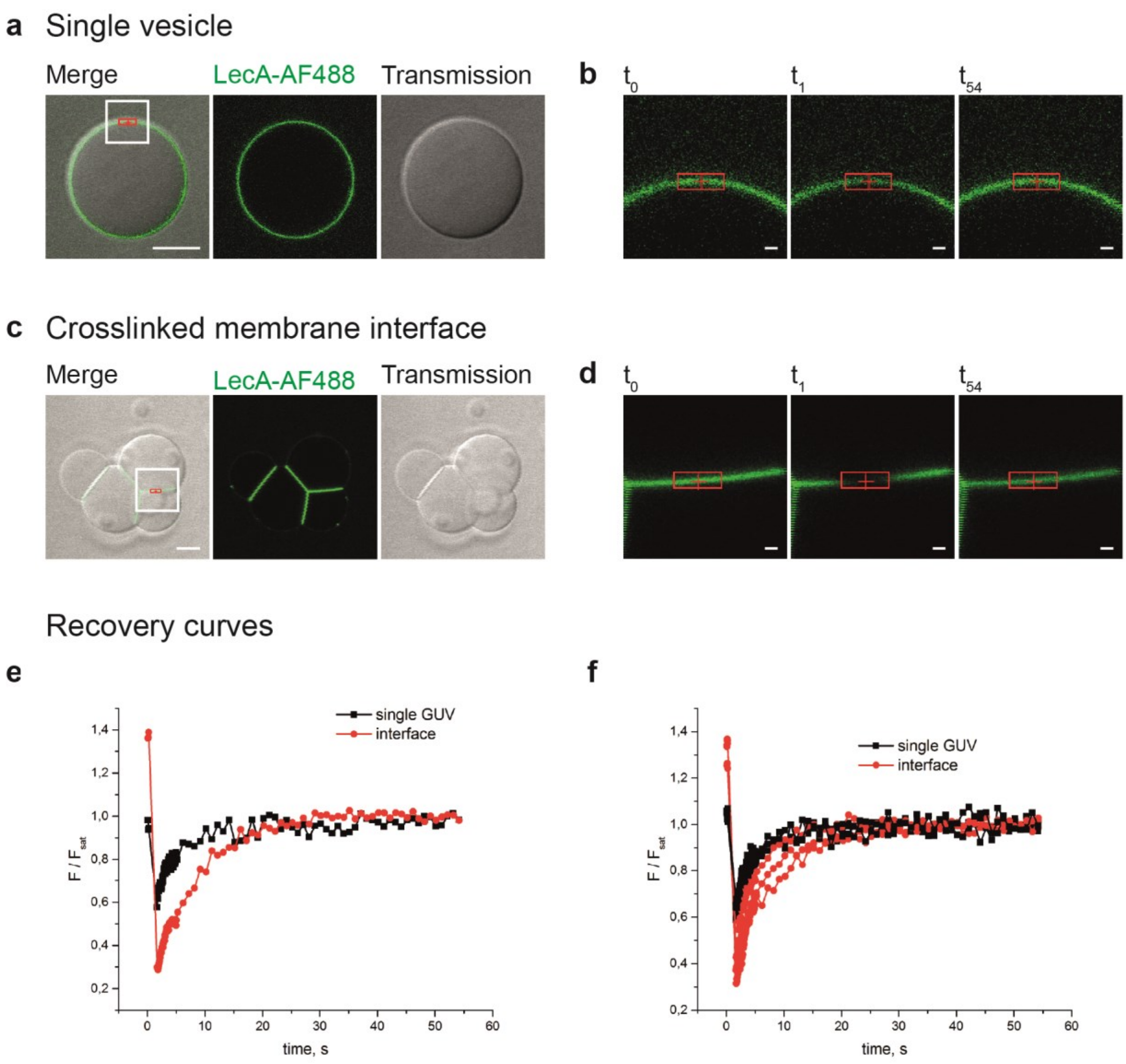

Supplementary Figure S3: LecA mobility on a single GUV surface and within interfaces studied by fluorescence recovery after photobleaching (FRAP)

GUVs composed of 65 mol% DOPC, 30 mol% cholesterol, and 5 mol% Gb3 were incubated with 100 nM LecA-AF488 (green) for >1 h. As no membrane dye was used a transmission image was included to visualise the membrane. (**a**) Image of a single vesicle with bound lectin used for FRAP. (**b**) Zoom of (a) showing the region of interest (ROI, red rectangle) before photobleaching (t_0), directly after photobleaching (t_1), and the last image of the FRAP experiment (t_{54}) where the recovery reached a saturation level. (**c**) Image of LecA crosslinked vesicles used for FRAP. (**d**) Zoom of (c) with the ROI (red rectangle) before photobleaching (t_0), directly after photobleaching (t_1), and after recovery (t_{54}). (**e**) Recovery curve of (b) with the mean fluorescence intensity (F) of the ROI normalized to the mean fluorescence intensity of saturation (F_{sat}) indicate that LecA diffuses faster on the GUV surface of single vesicles compared to within the contact area between vesicles. For single vesicles the membrane bound LecA diffused so fast that the intensity already recovered significantly before the

t_{1}-image was recorded. Furthermore, on single GUVs the fluorescence recovered almost to the pre-bleach level. In contrast, the fluorescence recovery of LecA within an interface was lower as here a considerable higher total amount of lectin was bleached. (**f**) Representative recovery curves of different vesicles illustrate that recovery dynamics of single GUVs are consistent, while they vary more for crosslinked GUVs due to differences in sizes and geometries of the individual interfaces. The scale bars in (a) and (c) are 10 µm; the scale bars in (b) and (d) are 1 µm.

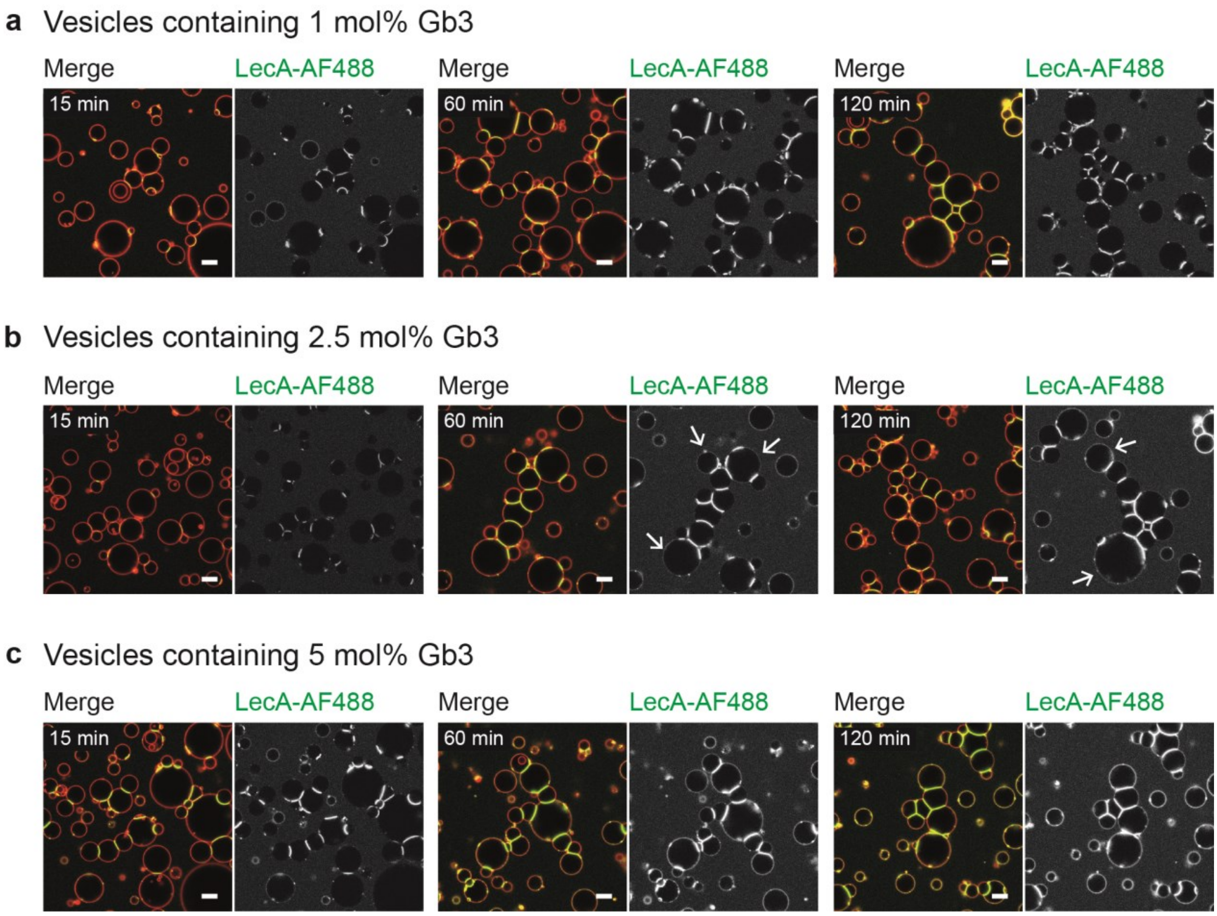

Supplementary Figure S4: Influence of Gb3 concentration on the binding and crosslinking behavior of LecA

GUVs were composed of 30 mol% cholesterol, 0.5 mol% DHPE-TxRed (red), and (**a**) 68.5 mol% DOPC for 1 mol% Gb3, (**b**) 67 mol% DOPC for 2.5 mol% Gb3, and (**c**) 64.5 mol% DOPC for 5 mol% Gb3, respectively. The TxRed channel (membrane dye) is depicted in the merge, but not as a separate image, as it does not provide additional information. The vesicles were incubated with 100 nM LecA-AF488 (green) for 2 h and a time series of 15 min, 60 min, and 120 min is depicted. (**a**) Vesicles with 1 mol% Gb3 exhibited no lectin accumulation outside of contact areas and weaker fluorescence intensities within interfaces. (**b**) Incorporation of 2.5 mol% Gb3 resulted in a more complex situation where mainly vesicles at the border of vesicle clusters (usually with a lower total-interface-area) showed lectin accumulation outside of contact regions (arrows). (**c**) GUVs with 5 mol% Gb3 appeared with a strong overall lectin binding, indicating that all interfaces were saturated with LecA bound to its ligand. Scale bars = 10 µm.

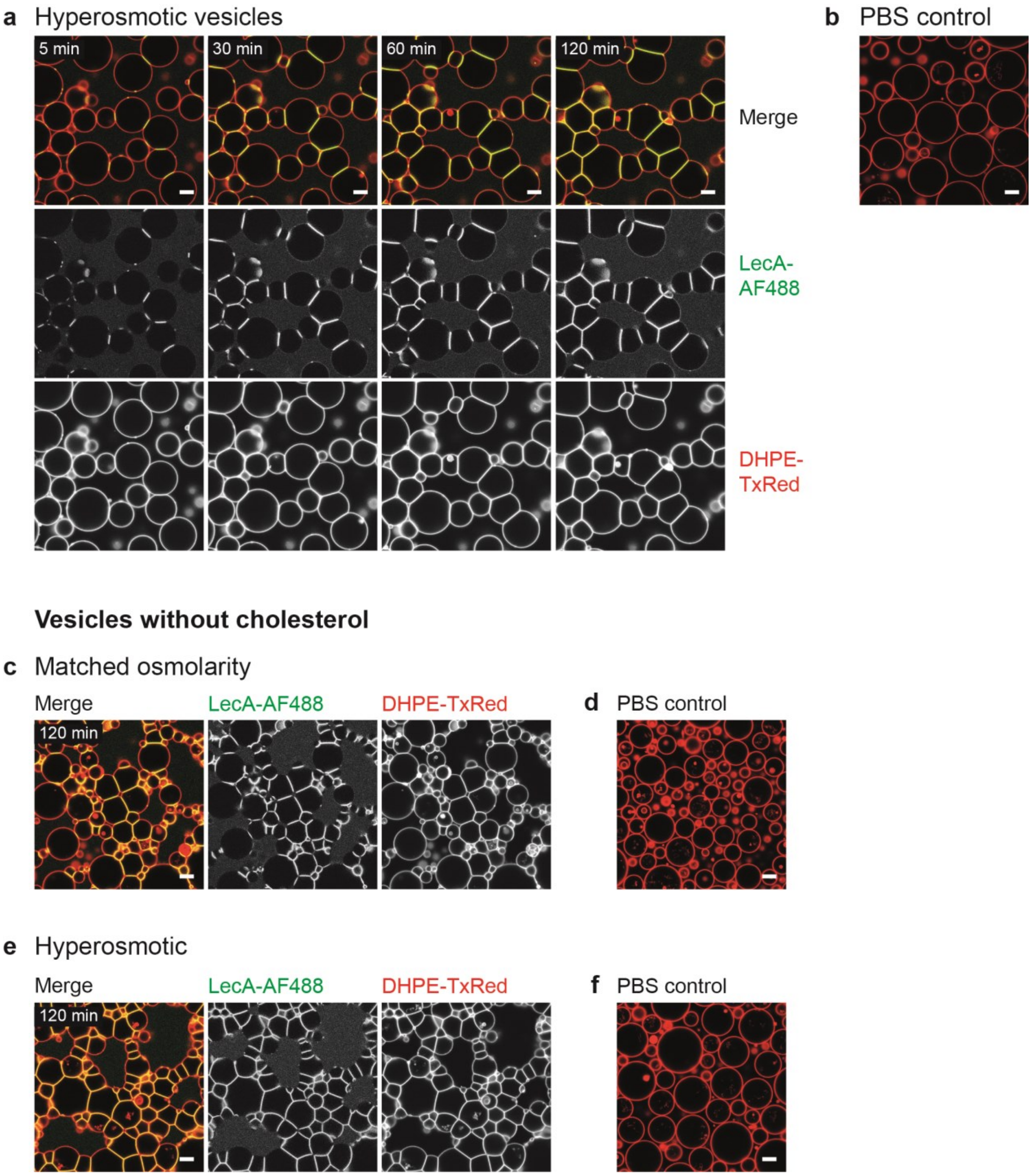

Supplementary Figure S5: Influence of osmolarity and lipid composition on the formation of protocellular junctions.

(**a**) Timelaps of protocell crosslinking with 100 nM LecA-AF488 (green) for indicated timepoints. Vesicles composed of DOPC:cholesterol:DHPE-TxRed (red) (64.5:30:0.5 mol%, respectively) and 5 mol% Gb3 were electroformated in hyperosmotic sucrose (~60 mOsm higher than the surrounding PBS). Vesicles did crosslink and lectin accumulation within contact areas resulted in the formation of elongated interfaces. (**b**) PBS mock control to (a). (**c-f**) Comparison to vesicles containing no cholesterol but 64.5 mol% DOPC, 0.5 mol% DHPE-TxRed and 5 mol% Gb3 with matched osmolarity

(c+d) or hyperosmotic sucrose (~60 mOsm higher than the surrounding PBS; e+f), where (d+f) show the PBS mock control. Despite the osmotic differences no significant discrepancies of vesicle crosslinking and formation of interfaces was observed. Scale bars are 10 μm.

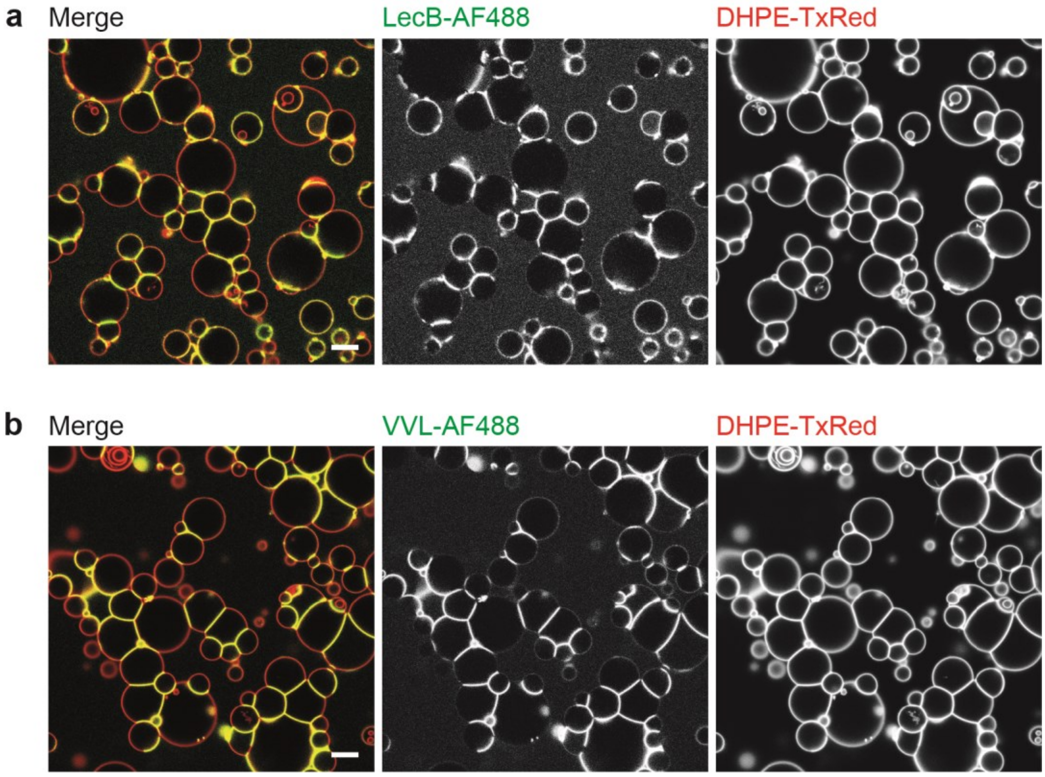

Supplementary Figure S6: Generation of protocellular junctions using LecB or VVL, two tetravalent lectins with opposing binding sites

(**a**) GUVs composed of DOPC:cholesterol:DHPE-TxRed (red) (64.5:30:0.5 mol%, respectively) and 5 mol% DOPE-Le^{a} were crosslinked with 100 nM LecB-AF488 (green) for >2 h. (**b**) GUVs composed of DOPC:cholesterol:DHPE-TxRed (red) (64.5:30:0.5 mol%, respectively) and 5 mol% CSG-peptide were crosslinked with 100 nM VVL-AF488 (green) for >2 h. (**a+b**) Vesicles were crosslinked by the respective lectins and presented the usual accumulation of lectin within interfaces, indicated by strong fluorescence within the latter. Scale bars = 10 µm.

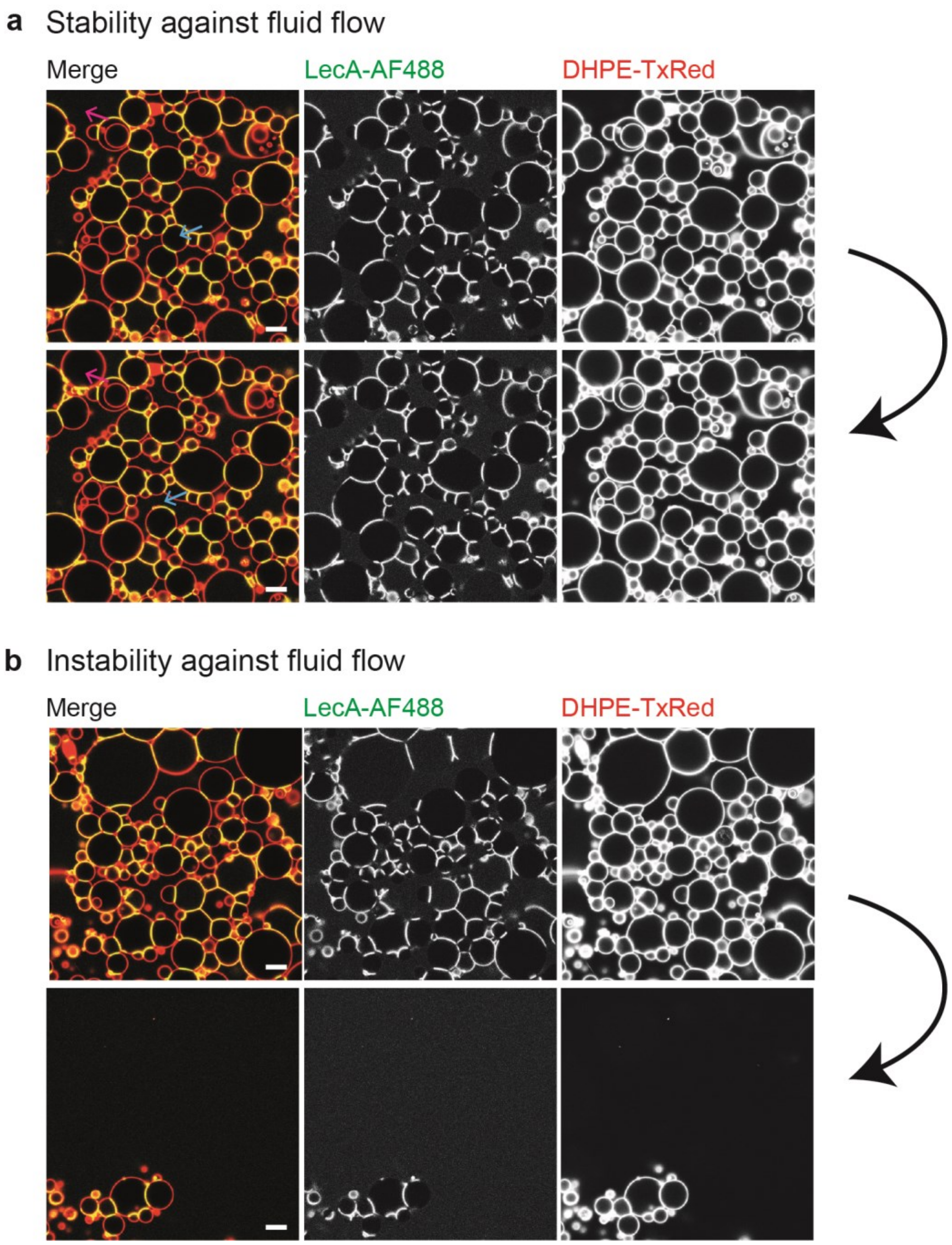

Supplementary Figure S7: Attachment to the support increases the stability against fluid flow

Chambers were coated with BSA-biotin followed by streptavidin. GUVs composed of DOPC:cholesterol:DHPE-TxRed (red):Gb3 (64 mol%:30 mol:0.5 mol:5 mol%, respectively) and 0.5 mol% DOPE-biotin were sedimented and given time to spread for 30 min prior to the addition of 100 nM LecA-AF488 (green) for >2 h. Vesicles were imaged before and after resuspension of three times one third of the chamber volume. (**a**) Representative image of vesicles which remained attached. Vesicles which disappear (blue arrow) or appear (pink arrow) illustrate small differences generated by resuspension. (**b**) Representative image of vesicles which were mostly flushed away by the fluid flow. Scale bars = 10 µm.

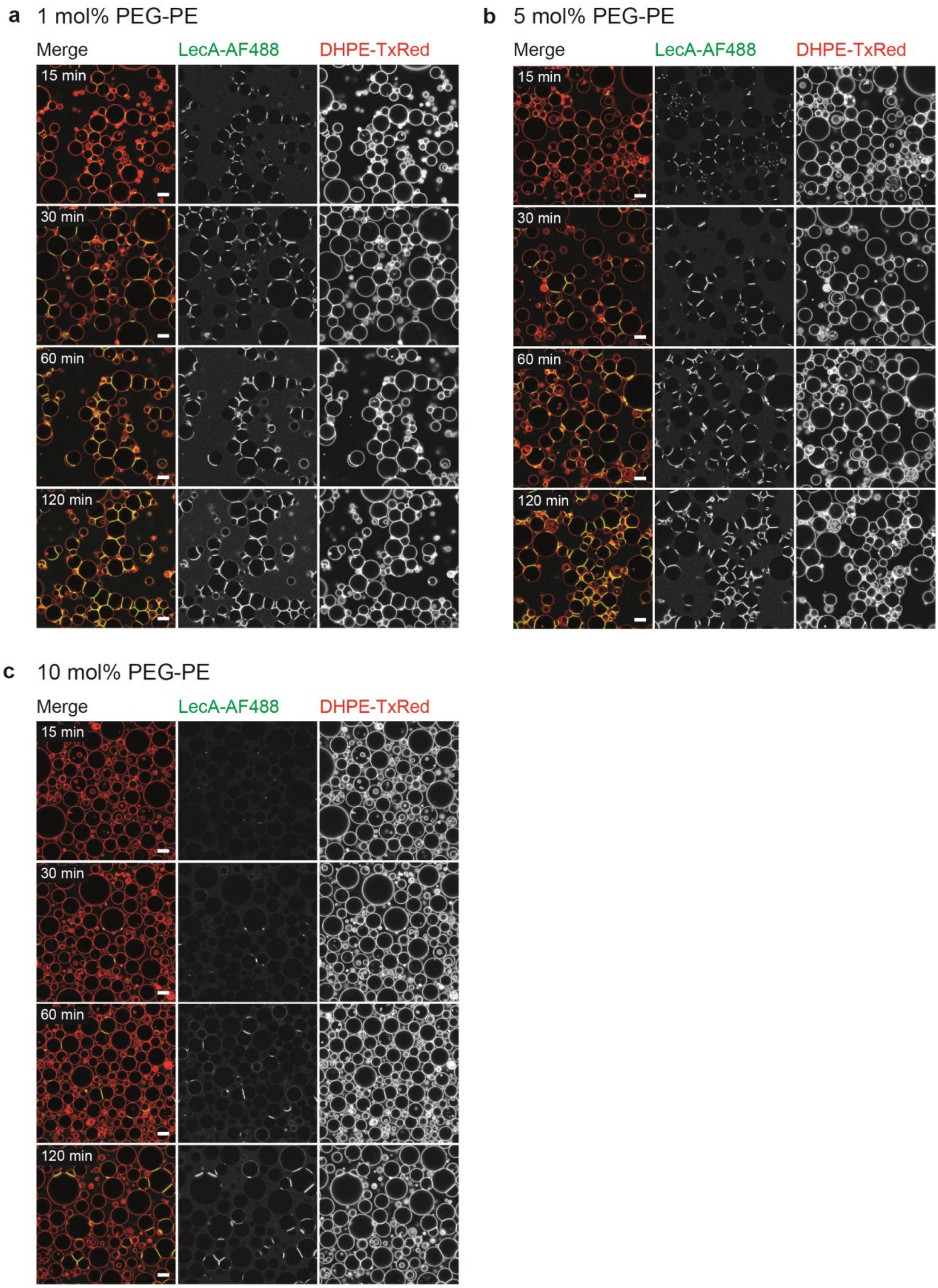

Supplementary Figure S8: Time series of lectin binding and the formation of protocellular junctions for different concentrations of PEG-modified lipids

100 nM LecA-AF488 was added to GUVs with differing amounts of the lipopolymer 18:0 PEG2000 PE referred to as PEG-PE. (**a**) LecA was accumulating in interfaces of GUVs composed of 62.4 mol% DOPC, 31.1 mol% cholesterol, 0.5 mol% DHPE-TxRed (red), 5 mol% Gb3, and 1 mol% PEG-PE, and protocellular junctions were formed as usual. In addition, lectin binding outside of interfaces was observed once the contact areas reached a saturation level. (**b**) GUVs with a lipid composition of 59.7 mol% DOPC, 29.8 mol% cholesterol, 0.5 mol% DHPE-TxRed, 5 mol% Gb3, and 5 mol% PEG-PE formed protocellular junctions despite the higher concentration of lipopolymer. However, no lectin binding outside of contact areas was observed. (**c**) GUVs were composed of 56.4 mol% DOPC, 28.1 mol% cholesterol, 0.5 mol% DHPE-TxRed, 5 mol% Gb3, and 10 mol% PEG-PE. Only a minority of vesicles showed lectin binding and formation of protocellular junctions although they were in close proximity, but with increased quantity over time. Scale bars = 10 µm.

Supplementary Movie S1: LecA crosslinking of vesicles containing Gb3, related to Fig. 1a

The white arrow indicates the accumulation of LecA-AF488 (green) within the contact area of two vesicles (membrane dye DHPE-TxRed, red) by an increase of fluorescence intensity and the grow in size of the interface. The pink arrow points towards the region of a membrane without contact to another vesicle, where the fluorescence intensity increases over time until 111 min, where the respective vesicle forms an additional interface to another GUV. Accordingly, the accumulation of fluorescence within the new contact zone is accompanied by a decrease of fluorescence outside the interface of the respective vesicle.
